# Supplementary material for: Generation and characterization of conditional yeast mutants affecting each of the 2 essential functions of the scaffolding proteins Boi1/2 and Bem1
Source: G3 (Bethesda). 2022 Oct 11;12(12):jkac273. doi: 10.1093/g3journal/jkac273 (PMC9713459; doi:10.1093/g3journal/jkac273)
Supplement: jkac273_Supplementary_Figure_S1 [file jkac273_supplementary_figure_s1.pdf]

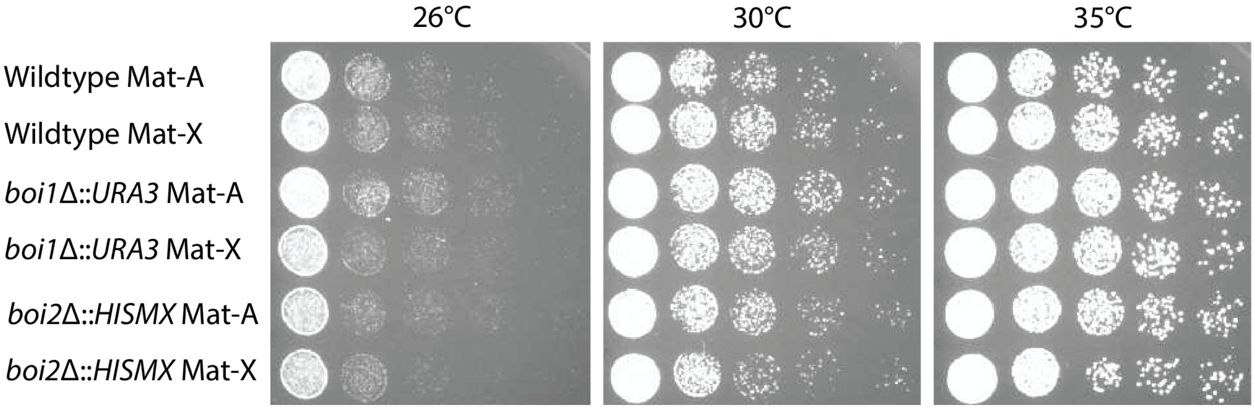

Supplemental Figure 1.1: *boi1*Δ or *boi2*Δ growth. 10-fold dilution assays of wildtype, *boi1Δ::URA3*, and *boi2Δ::HISMX* of each mating type at three temperatures: 26°C, 30°C, and 35°C on YPD for 24 hours.

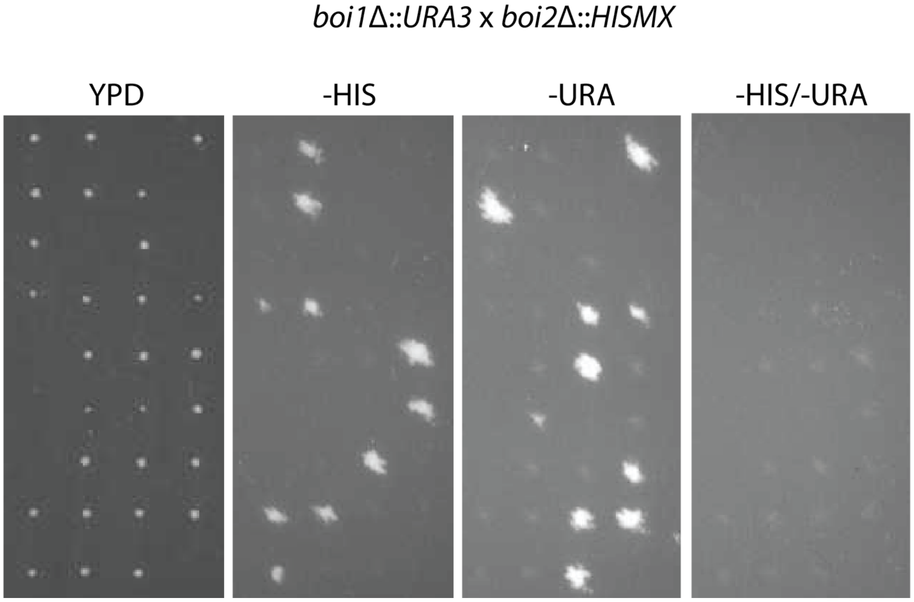

Supplemental Figure 1.2: Either Boi1 or Boi2 is essential for cell survival in the BY4741/2 background. Tetrad dissections of mated cells: *boi1Δ::URA3* x *boi2Δ::HISMX* diploid cells on YPD. Tetrads were replica plated on -HIS to select for *boi2Δ::HISMX*, -URA to select for *boi1Δ::URA3*, and -HIS/-URA to select for *boi1Δ::URA3 boi2Δ::HISMX*. There were no viable *boi1Δ::URA3 boi2Δ::HISMX* spores. All plates were grown for 2 days at 26°C.
