# Supplementary material for: Generation and characterization of conditional yeast mutants affecting each of the 2 essential functions of the scaffolding proteins Boi1/2 and Bem1
Source: G3 (Bethesda). 2022 Oct 11;12(12):jkac273. doi: 10.1093/g3journal/jkac273 (PMC9713459; doi:10.1093/g3journal/jkac273)
Supplement: jkac273_Supplementary_Figure_S2 [file jkac273_supplementary_figure_s2.pdf]

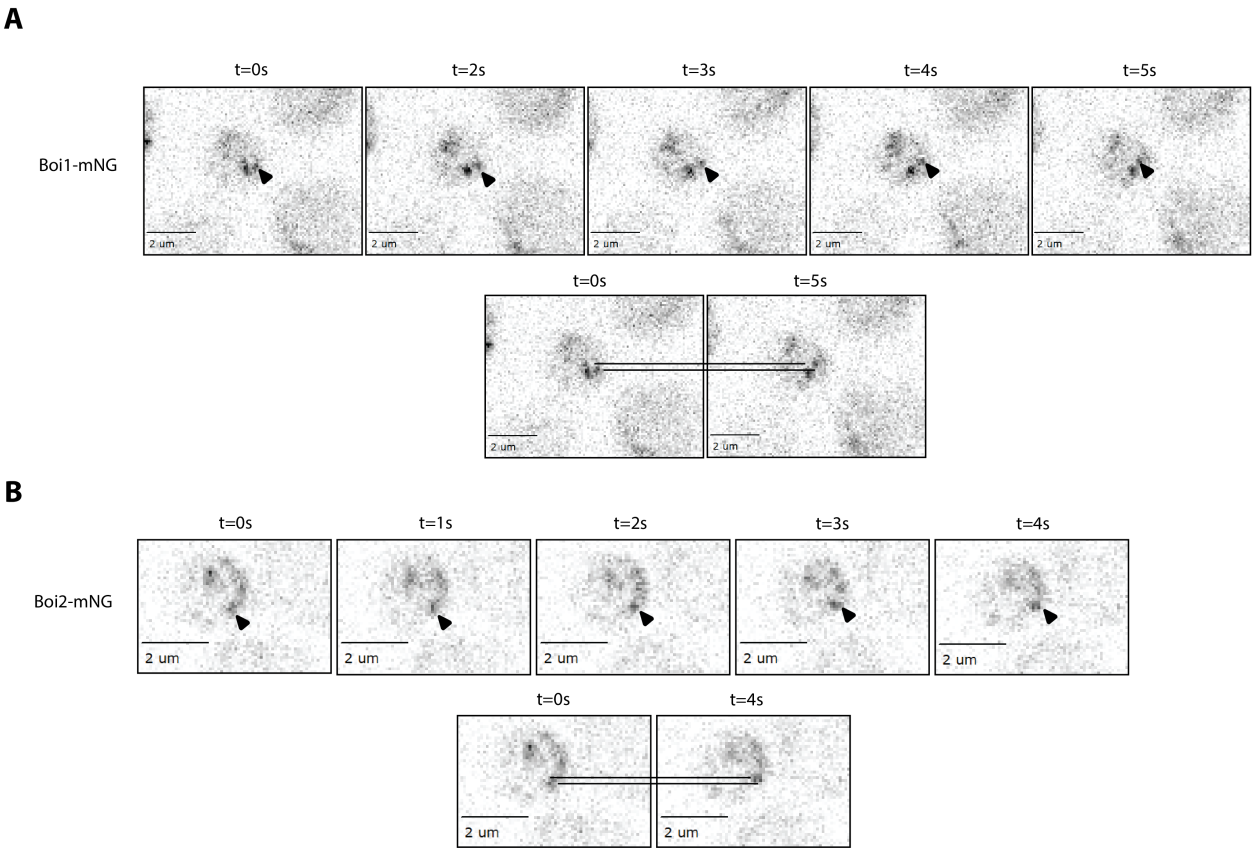

Supplemental 2.1: Lateral movement of Boi1-mNG and Boi2-mNG along the bud cortex. (A) Example of lateral patch movement in Boi1-mNG taken from videos similar to Video 1 with 50ms exposure timelapse captured every 160ms. Patch moves upwards along the cortex (highlighted with black arrows), slightly. Lower panel shows the displacement of the patch from the first timepoint to the last. (B) Example of lateral patch movement in Boi2-mNG taken with the same parameters as in A.
