# Supplementary material for: Generation and characterization of conditional yeast mutants affecting each of the 2 essential functions of the scaffolding proteins Boi1/2 and Bem1
Source: G3 (Bethesda). 2022 Oct 11;12(12):jkac273. doi: 10.1093/g3journal/jkac273 (PMC9713459; doi:10.1093/g3journal/jkac273)
Supplement: jkac273_Supplementary_Figure_S3 [file jkac273_supplementary_figure_s3.pdf]

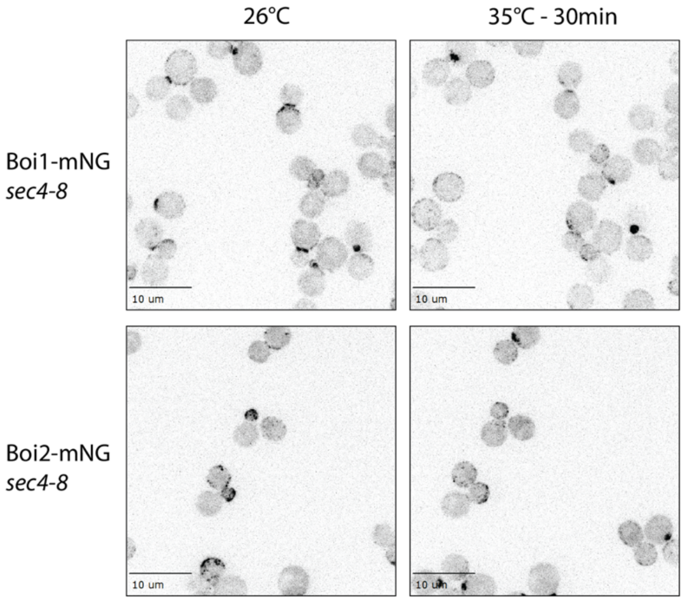

Supplemental Figure 3.1: Boi1-mNG and Boi2-mNG in *sec4-8* cells. Boi1-mNG and Boi2-mNG in *sec4-8* cells either at 26°C or shifted to the restrictive temperature, 35°C for 30 minutes on the CherryTemp. Cells were imaged with 15 plane z-stacks and were exposed for 200ms. Scale bars are 10µm.

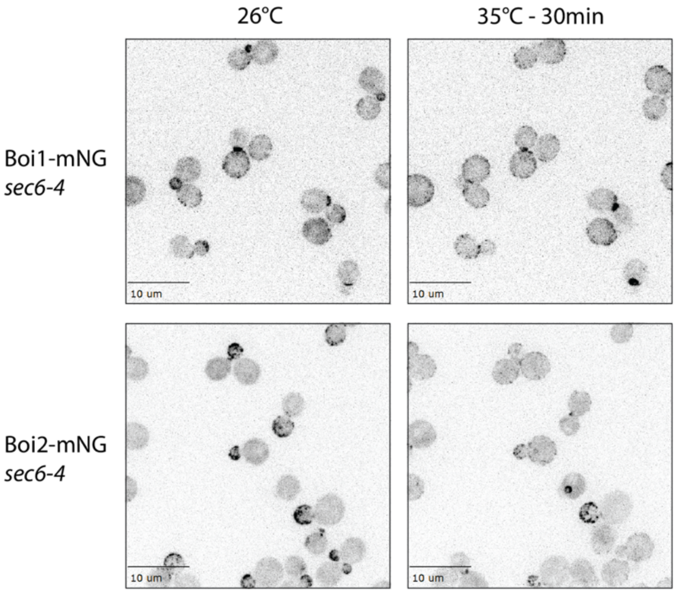

Supplemental Figure 3.2: Boi1-mNG and Boi2-mNG in *sec6-4* cells. Boi1-mNG and Boi2-mNG in *sec6-4* cells either at 26°C or shifted to the restrictive temperature, 35°C for 30 minutes on the CherryTemp. Cells were imaged with 15 plane z-stacks and were exposed for 200ms. Scale bars are 10µm.

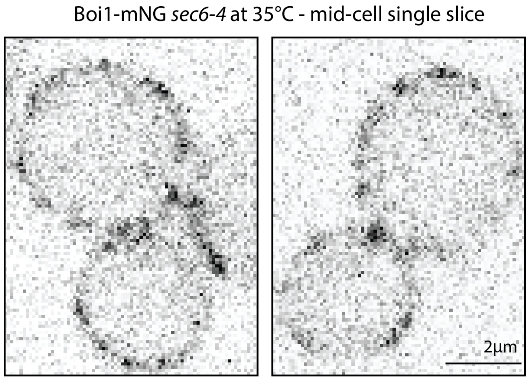

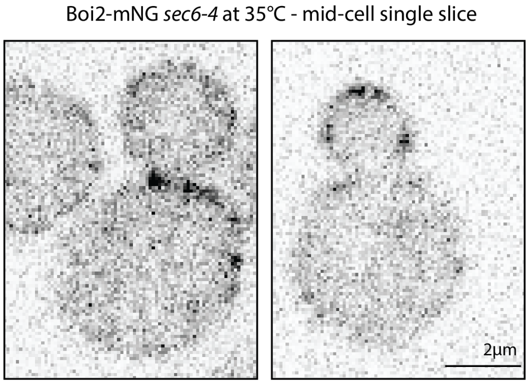

Supplemental Figure 3.3: Single slice images from Supplemental Figure 2.2 through the middle of the cell. Boi1-mNG and Boi2-mNG in *sec6-4* cells shifted to 35°C for over 30 minutes on the Cherry Temp. Exposure of 200ms. Scale bars are 2µm.
