# Supplementary material for: Generation and characterization of conditional yeast mutants affecting each of the 2 essential functions of the scaffolding proteins Boi1/2 and Bem1
Source: G3 (Bethesda). 2022 Oct 11;12(12):jkac273. doi: 10.1093/g3journal/jkac273 (PMC9713459; doi:10.1093/g3journal/jkac273)
Supplement: jkac273_Supplementary_Figure_S4 [file jkac273_supplementary_figure_s4.pdf]

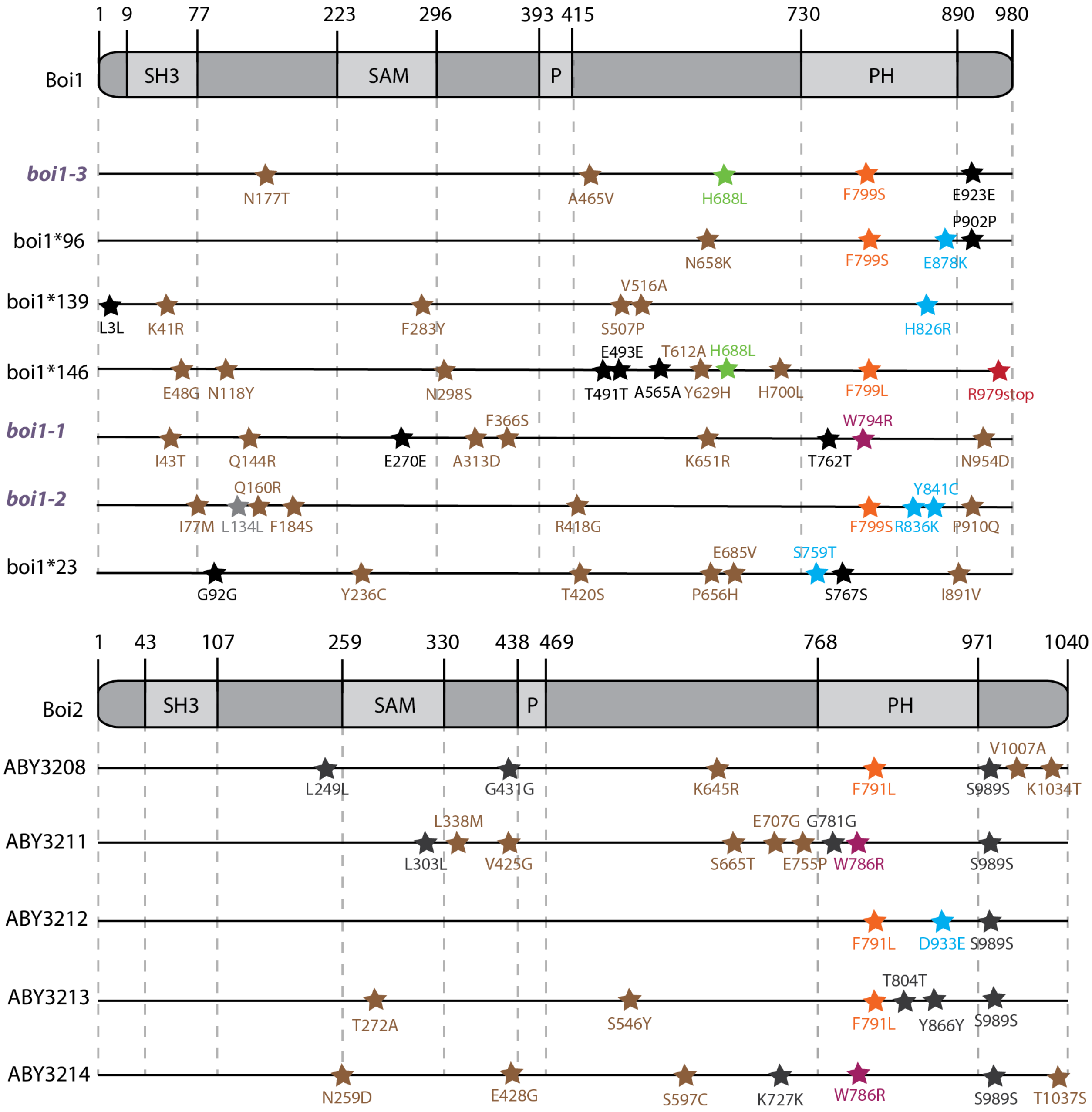

Supplemental Figure 4.1: Mutations in the temperature sensitive candidates in *BOI1* or *BOI2* mutants. Mutations found throughout *BOI1* and *BOI2* in several candidates along with the chosen temperature sensitive mutants (*boi1-1, boi1-2*, and *boi1-3*, highlighted in purple). Interesting PH domain mutations are highlighted in blue and green, along with missense mutations in brown and silent mutations in black to show the mutational coverage of the different screens.

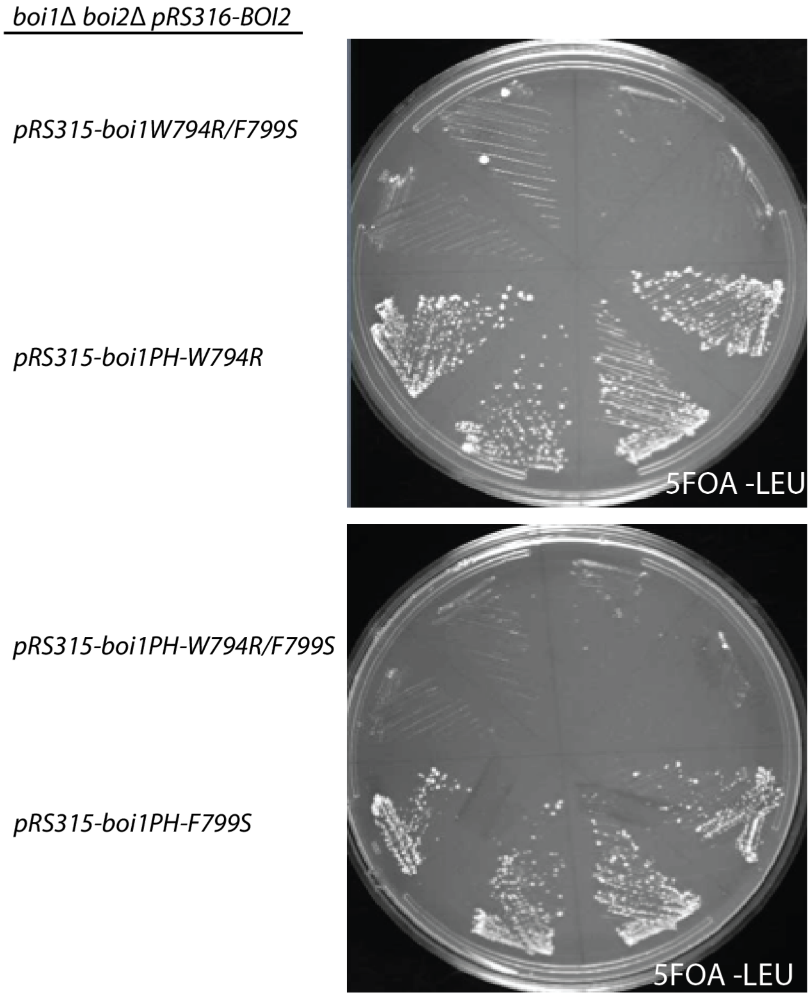

Supplemental Figure 4.2: Double mutants of *boi1W794R/F799S* are inviable. Plates showing four isolates with pRS315 plasmids carrying*: boi1W794R/F799S, boi1PH-W794R, boi1PH-W794R/F799S*, or *boi1PH-799S*. Plasmids were transformed into *boi1Δ boi2Δ pRS316-BOI2* cells and then plated on 5FOA -LEU to select against pRS316-BOI2 plasmid. Cells were grown for 3 days at 26°C.

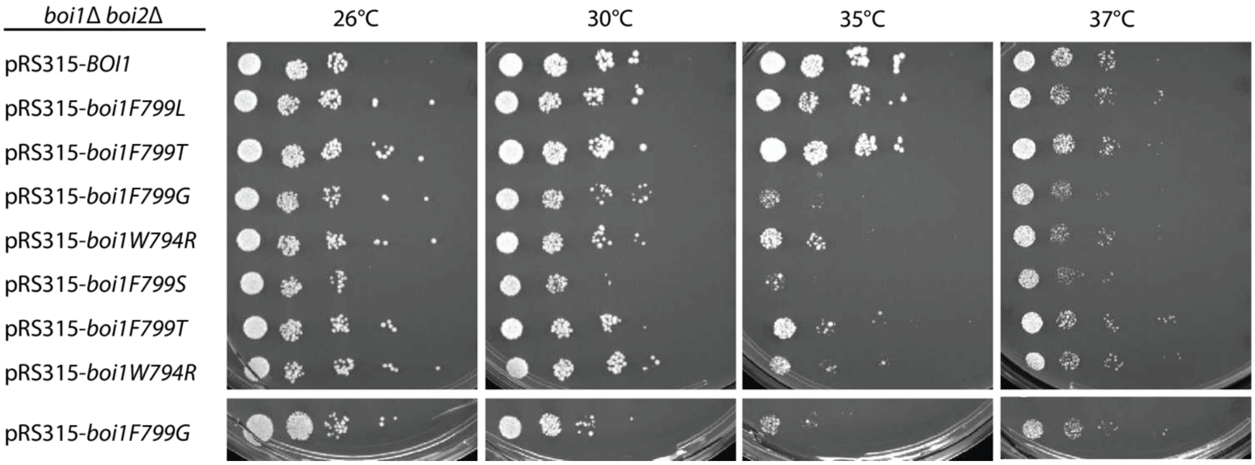

Supplemental Figure 4.3: Mini-screen of *Boi1W794X* and *boi1F799X* show that these positions are critical for function. Plates showing viable candidates from the screens looking at Boi1W794 and Boi1F799 mutated to virtually every other amino acid possible in yeast. The most sensitive candidates are: *Boi1W794R, Boi1W794G, Boi1F799S*, and *Boi1F799G*. Plates were grown at their respective temperatures for 2 days on SC -LEU.
