# Supplementary material for: Generation and characterization of conditional yeast mutants affecting each of the 2 essential functions of the scaffolding proteins Boi1/2 and Bem1
Source: G3 (Bethesda). 2022 Oct 11;12(12):jkac273. doi: 10.1093/g3journal/jkac273 (PMC9713459; doi:10.1093/g3journal/jkac273)
Supplement: jkac273_Supplementary_Figure_S5 [file jkac273_supplementary_figure_s5.pdf]

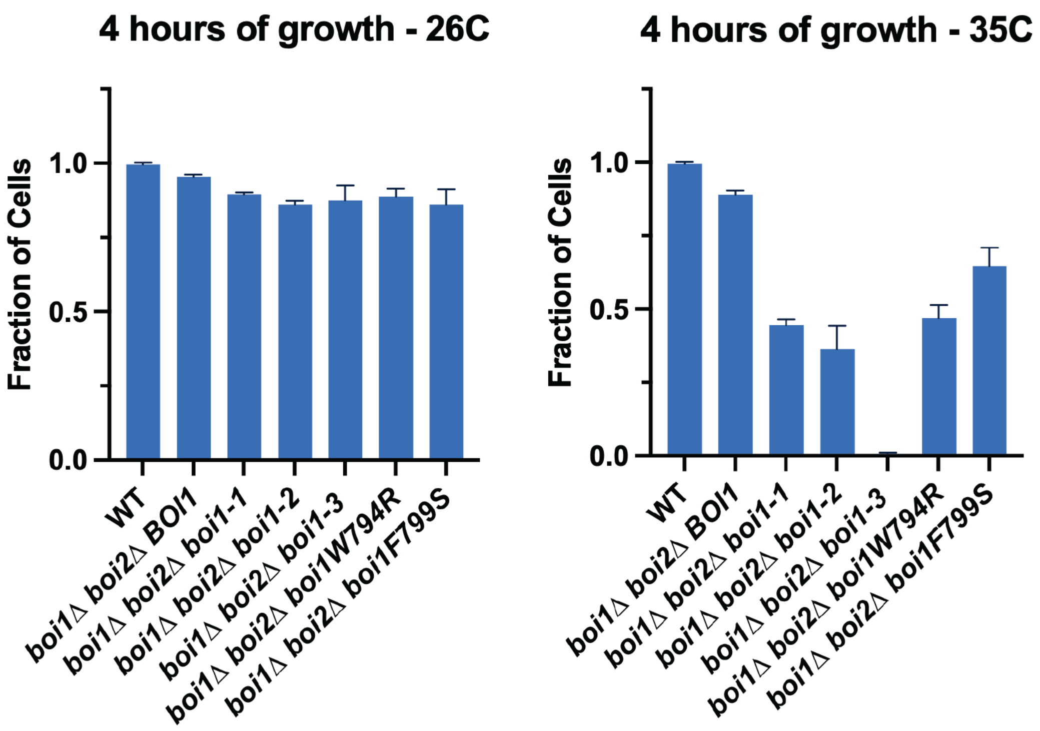

Supplemental Figure 5.1: Percentage of viable cells alive after incubation at respective temperatures. Data shows percentage of viable cells alive at the end of a 4-hour time-period at the respective temperatures. Data was collected by observing cells in at least 5 different fields of view with at minimum n=100. Graphs are a fraction of viable cells analyzed in each cell type.

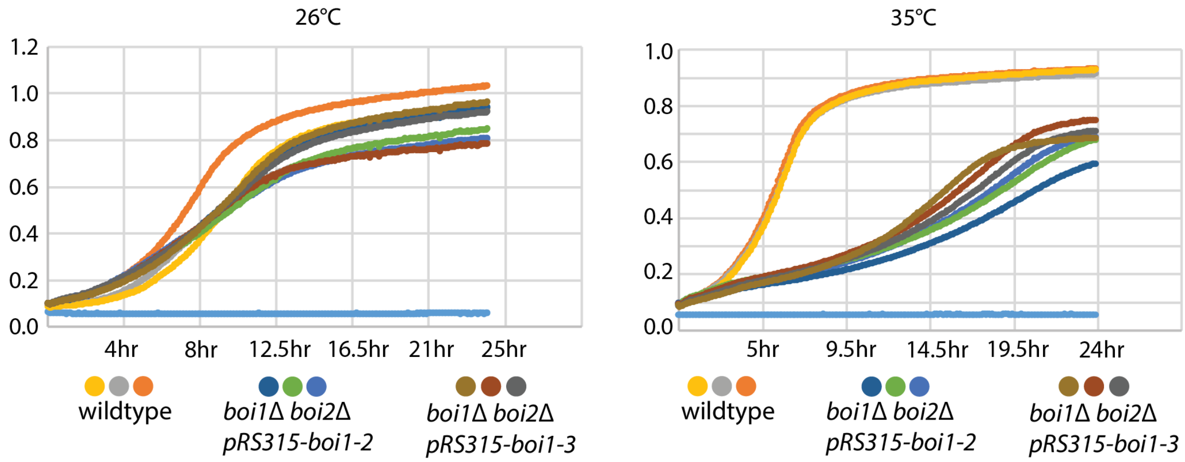

Supplemental Figure 5.2: Growth curves of *boi1* mutants compared to wildtype at permissive and restrictive temperatures. Graphs show optical density at 600nm of the cells grown at 26°C and at 35°C - three replicates of each cell type are shown including a blank (shown in cyan).
