# Supplementary material for: Generation and characterization of conditional yeast mutants affecting each of the 2 essential functions of the scaffolding proteins Boi1/2 and Bem1
Source: G3 (Bethesda). 2022 Oct 11;12(12):jkac273. doi: 10.1093/g3journal/jkac273 (PMC9713459; doi:10.1093/g3journal/jkac273)
Supplement: jkac273_Supplementary_Figure_S6 [file jkac273_supplementary_figure_s6.pdf]

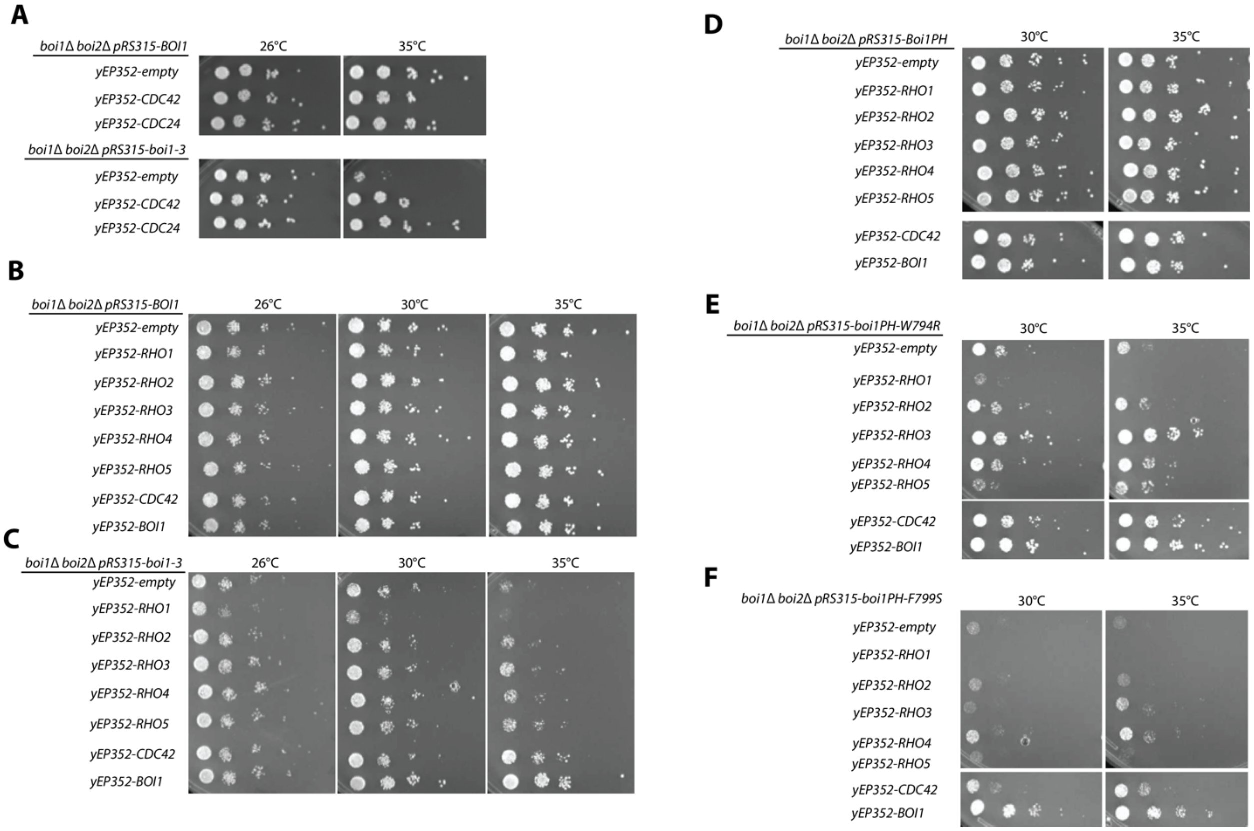

Supplemental Figure 6.1: Suppression of boi1 conditional mutants by over-expression of the Cdc42 pathway. (A) Growth assays of wildtype *BOI1* or *boi1-3* in a *boi1Δ boi2Δ* cells carrying yEP352 plasmids with either an empty plasmid, *CDC42*, or the GAP *CDC24*. Cells were grown on SC -URA/-LEU media for 2 days. (B and C) Growth assays of wildtype *BOI1* or *boi1-3* in a *boi1Δ boi2Δ* background with yEP352 plasmids either empty or with: *RHO1, RHO2, RHO3, RHO4, RHO5, CDC42*, or *BOI1*. Cells were grown on SC-URA/-LEU media for 2 days. (D, E, and F) Growth of *boi1Δ boi2Δ* background cells with a pRS315 vector containing Boi1PH (Boi1-730-980 C-terminal region) (D), *boi1PH-W794R* (E), or *boi1PH-F799S* (F), with the addition of yEP352 vector empty, or with the indicated Rho GTPases, similar to panels B and C. Cells were plated on SC -URA/-LEU and grown at respective temperatures for 2 days.

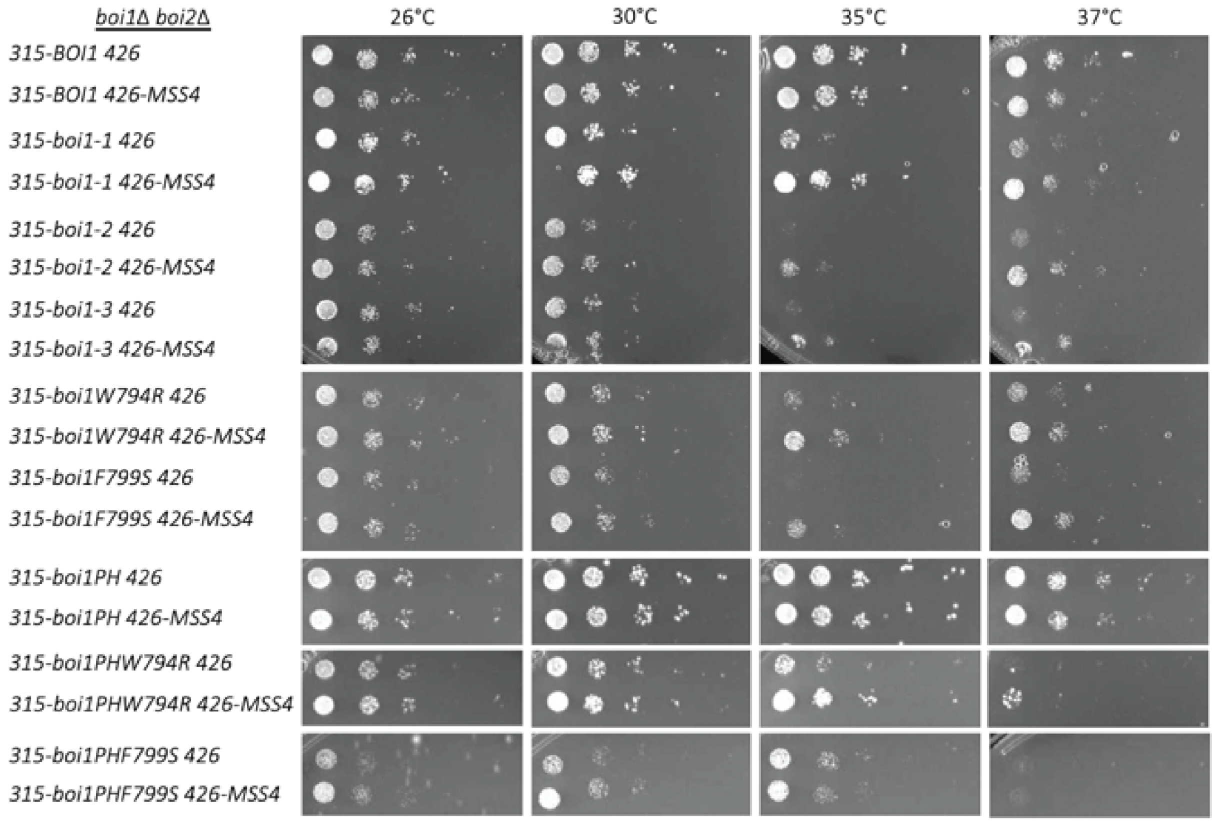

Supplemental Figure 6.2: Growth of *BOI1* mutants upon over-expression of *MSS4*. *MSS4* overexpressed with pRS426 2µm plasmid transformed into each of the indicated mutants. Dilution assays plated on -LEU/-URA plates and incubated at their respective temperatures for 2 days.
