# Supplementary figures and images for: Generation and characterization of conditional yeast mutants affecting each of the 2 essential functions of the scaffolding proteins Boi1/2 and Bem1
Source: G3 (Bethesda). 2022 Oct 11;12(12):jkac273. doi: 10.1093/g3journal/jkac273 (PMC9713459; doi:10.1093/g3journal/jkac273)

**A**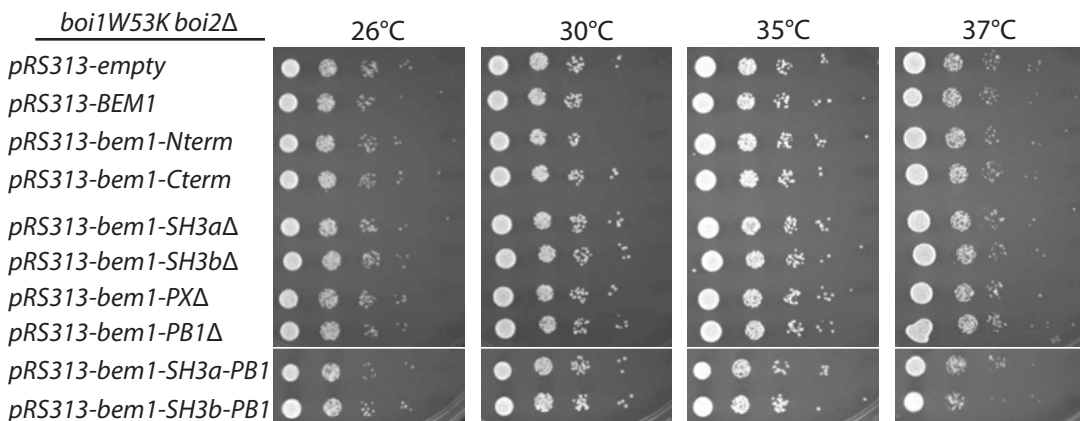**B**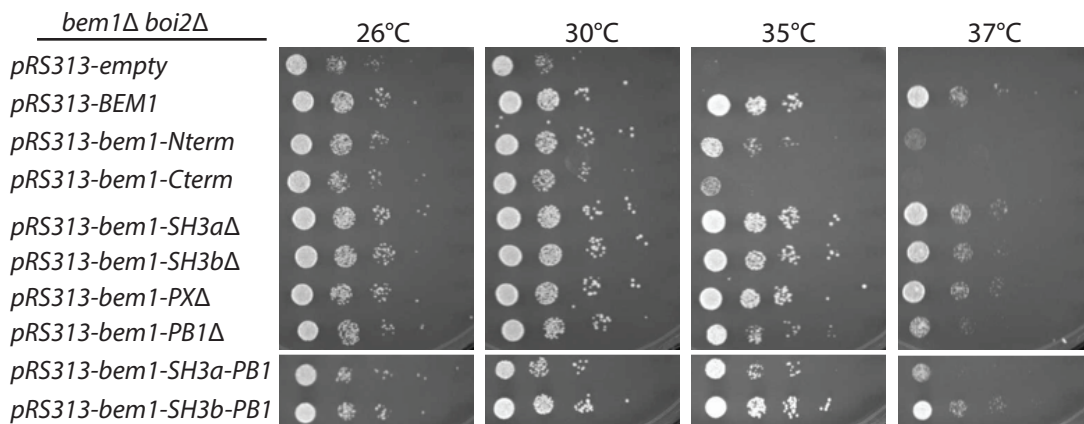**C**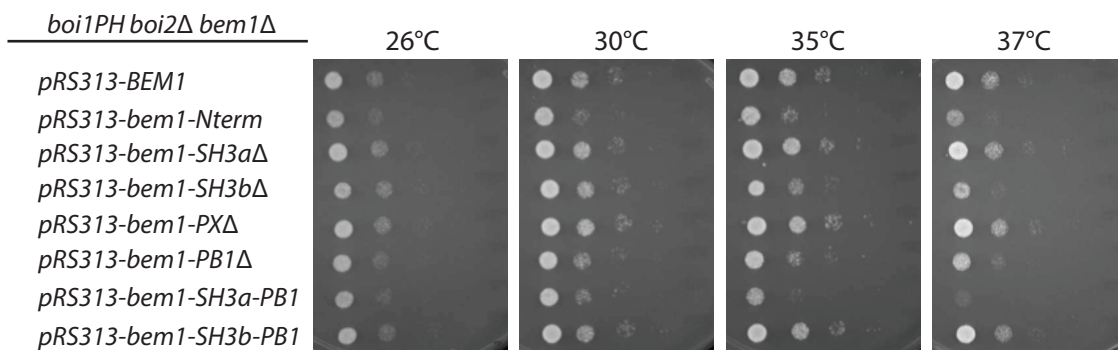

Supplement: jkac273_Supplementary_Figure_S7 [file jkac273_supplementary_figure_s7.pdf]
