# Supplementary material for: Generation and characterization of conditional yeast mutants affecting each of the 2 essential functions of the scaffolding proteins Boi1/2 and Bem1
Source: G3 (Bethesda). 2022 Oct 11;12(12):jkac273. doi: 10.1093/g3journal/jkac273 (PMC9713459; doi:10.1093/g3journal/jkac273)
Supplement: jkac273_Supplementary_Figure_S8 [file jkac273_supplementary_figure_s8.pdf]

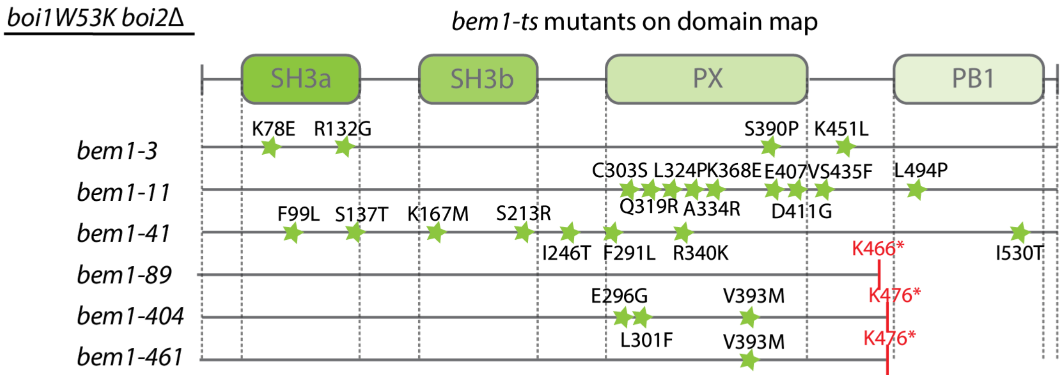

Supplemental Figure 8.1: Location of mutations in temperature sensitive candidates of *BEM1*. Termination codons are indicated in red by the preceding amino acid residue.

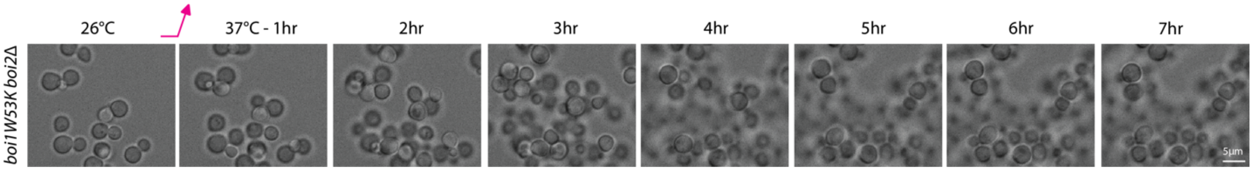

Supplemental Figure 8.2: DIC images of *boi1W53K boi2Δ* cells during growth. Cells were imaged once every five minutes for several hours after shifting to the restrictive temperature of 37°C. Scale bars are 5μm.
